# Anemia in children aged 6–59 months was significantly associated with maternal anemia status in rural Zimbabwe

**DOI:** 10.1002/fsn3.3157

**Published:** 2022-12-05

**Authors:** Beaula Mutonhodza, Mavis P. Dembedza, Murray R. Lark, Edward J. M. Joy, Muneta G. Manzeke‐Kangara, Handrea Njovo, Tasiana K. Nyadzayo, Alexander A. Kalimbira, Elizabeth H. Bailey, Martin R. Broadley, Tonderayi M. Matsungo, Prosper Chopera

**Affiliations:** ^1^ Department of Nutrition, Dietetics and Food Sciences University of Zimbabwe Harare Zimbabwe; ^2^ School of Biosciences, Sutton Bonington Campus University of Nottingham Loughborough, Leicestershire UK; ^3^ London School for Hygiene & Tropical Medicine London UK; ^4^ Rothamsted Research Harpenden UK; ^5^ National Nutrition Unit Ministry of Health and Child Care of Zimbabwe Harare Zimbabwe; ^6^ Department of Human Nutrition and Health Lilongwe University of Agriculture and Natural Resources Lilongwe Malawi

**Keywords:** anemia, micronutrient supplementation, stunting, water sanitation and hygiene, Zimbabwe

## Abstract

Globally, anemia is a public health problem affecting mostly women of reproductive age (WRA, *n* = 452) and children aged 6–59 months (*n* = 452) from low‐ and lower‐middle‐income countries. This cross‐sectional study assessed the prevalence and determinants of anemia in WRA and children aged 6–59 months in rural Zimbabwe. The venous blood sample was measured for hemoglobin utilizing a HemoCue machine. Anthropometric indices were assessed and classified based on World Health Organization standards. Socioeconomic characteristics were assessed. The median (±inter quartile range (IQR)) age of WRA was 29 ± 12 years and that for children was 29 ± 14 months. The prevalence of anemia was 29.6% and 17.9% in children and WRA, respectively, while the median (±IQR) hemoglobin levels were 13.4 ± 1.8 and 11.7 ± 1.5 g/dl among women and children, respectively. Multiple logistic regression analysis was used to assess determinants of anemia. Anemia in children was significantly associated with maternal anemia (odds ratio (OR) = 2.02; 95% CI 1.21–3.37; *p* = .007) and being a boy (OR = 0.63; 95% CI 0.41–0.95; *p* = .029), while anemia in WRA was significantly associated with the use of unimproved dug wells as a source of drinking water (OR = 0.36; 95% CI 0.20–0.66; *p* = .001) and lack of agricultural land ownership (OR = 0.51; 95% CI 0.31–0.85; *p* = .009). Anemia is a public health problem in the study setting. The positive association between maternal and child anemia reflects the possibility of cross‐generational anemia. Therefore, interventions that focus on improving preconceptual and maternal nutritional status may help to reduce anemia in low‐income settings.

## Graphical abstract

Anaemia is of moderate public health significance in children and of mild public health significance in women of reproductive age. Positive association between maternal and child anaemia reflects possibility of cross‐generational origins of anaemia. Anaemia prevalence higher in boys compared to girls.

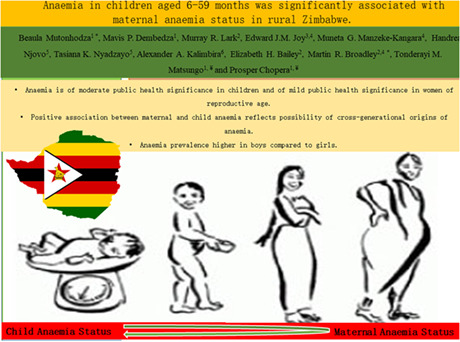

## 1 INTRODUCTION

Anemia is a condition in which the number of red blood cells and consequently their oxygen‐carrying capacity is insufficient to meet the body's physiologic oxygen needs (WHO & Chan, 2011; WHO, 2020a). Anemia affects approximately one‐quarter of the world's population and is concentrated in preschool children (PSC) and women of reproductive age (WRA) (McLean et al., 2009). Causes of anemia include blood loss, infections, acute and chronic diseases, micronutrient deficiencies, splenomegaly, and hemoglobinopathies (Janus & Moerschel, 2010; Elmardi et al., 2020). However, available evidence shows that iron deficiency (ID) is the most common cause of anemia (Camaschella, 2017; WHO, 2017) contributing to approximately 50% of the anemia cases worldwide (WHO, 2012). However, the situation will be lower in settings with high rates of inflammation and infection (Petry et al., 2016). The current study looked at anemia defined as low hemoglobin (Hb) levels, Hb < 11 g/dl for children and Hb < 12 g/dl for WRA. Anemia is associated with adverse reproductive outcomes such as preterm delivery, low‐birth‐weight infants, and decreased iron stores for the baby, which leads to impaired health and productivity later in life (Black et al., 2008). Failure to reduce anemia may result in maternal and neonatal deaths, a combination that accounts for 2.5–3.4 million deaths worldwide (WHO & Worldbank, 2010).

Anemia is an indicator of both poor nutrition and poor health (WHO, 2021). Women of reproductive age and children are deemed the most vulnerable to micronutrient deficiencies due to greater physiological requirements (Ahmed et al., 2017). In 2019, the global anemia prevalence was 29.9% in WRA, equivalent to over half a billion women aged 15–49 years, and 39.8% in children aged 6–59 months, equivalent to 269 million children with anemia. The prevalence of anemia in children under 5 years of age was highest in the African Region (WHO, 2021). According to the 2015 Zimbabwe Demographic Health Survey (ZDHS), more than a third of children aged 6–59 months (37%) were anemic while 27% of WRA had anemia (ZIMSTAT, 2015).

Anemia is often associated with multiple proximal risk factors (iron and vitamin A deficiencies, inflammation, malaria, age, gender, and body mass index [BMI]) and distal risk factors (education status, sanitation and hygiene facilities, and urban or rural residence) (Cane et al., 2022; Mrimi et al., 2022; Wirth et al., 2017). In Zimbabwe, the major causes of anemia in children and WRA are likely to include malaria, helminths, iron, and other nutritional deficiencies and chronic infections (ZIMSTAT, 2015). Zimbabwe does not have any recent national‐level statistics on the prevalence and predictors of anemia after the ZDHS 2015 survey. Furthermore, the emergence of the COVID‐19 pandemic resulted in restricted mobility and isolation, reduced income, and reduced access to essential health and nutrition services, and these dynamics are likely to have exacerbated malnutrition and food insecurity. Hence, anemia prevalence, as well as predictors, may have changed and thus highlights the need for studies to summarize the evolved scenario in the context of evidence‐based health delivery.

The current study was designed to explore demographic, health, and nutritional characteristics associated with anemia among WRA and children aged 6–59 months in selected districts in rural Zimbabwe. The data for the present study were collected as part of the baseline for a micronutrient biomarker survey “Translating GeoNutrition (TGN): Reducing mineral micronutrient deficiencies (MMNDs) in Zimbabwe (Grant Ref: EP/T015667/1).”

## 2 MATERIALS AND METHODS

### 2.1 Study site, sampling, and participants

The cross‐sectional study presents data on the prevalence and determinants of anemia in WRA and children aged 6–59 months from two rural districts Shamva (17.04409°S, 31.6739°E) and Mutasa (18.6155°S, 32.6730°E) in Zimbabwe. Data collection was done between December 2021 and January 2022. The sampling design was nested at the level of the national Demographic Health Survey (DHS) sampling approach (ZIMSTAT, 2015);

### 2.2 Primary sampling units (PSU)

The first stage of the sampling frame was made up of 30 enumeration areas (EA) selected per district, each EA had a unique 10‐digit geo‐code that reflected the province, district, ward, and land‐use sector in which it was located. EAs were selected by proportional probability sampling (PPS) with inclusion probabilities proportional to the most recently recorded population (ZIMSTAT, 2012). Maps for all selected EAs were provided by Zimbabwe Statistics Agency (ZIMSTAT) for EA identification. The selection of EAs was done using the formula:

$$\mathrm{Phi}=\sum \frac{\text{AhMhi}}{\mathrm{Mhi}},$$

where:

- *Phi* is the selection probability for EA number *i* in stratum *h*
- *Ah* is the number of EAs selected in stratum *h*
- *Mhi* is the number of households in EA number *hi* according to the population census
- *∑Mhi* is the number of households in stratum *h* according to the population census

### 2.3 Secondary sampling units (SSU)

**FIGURE 1:**
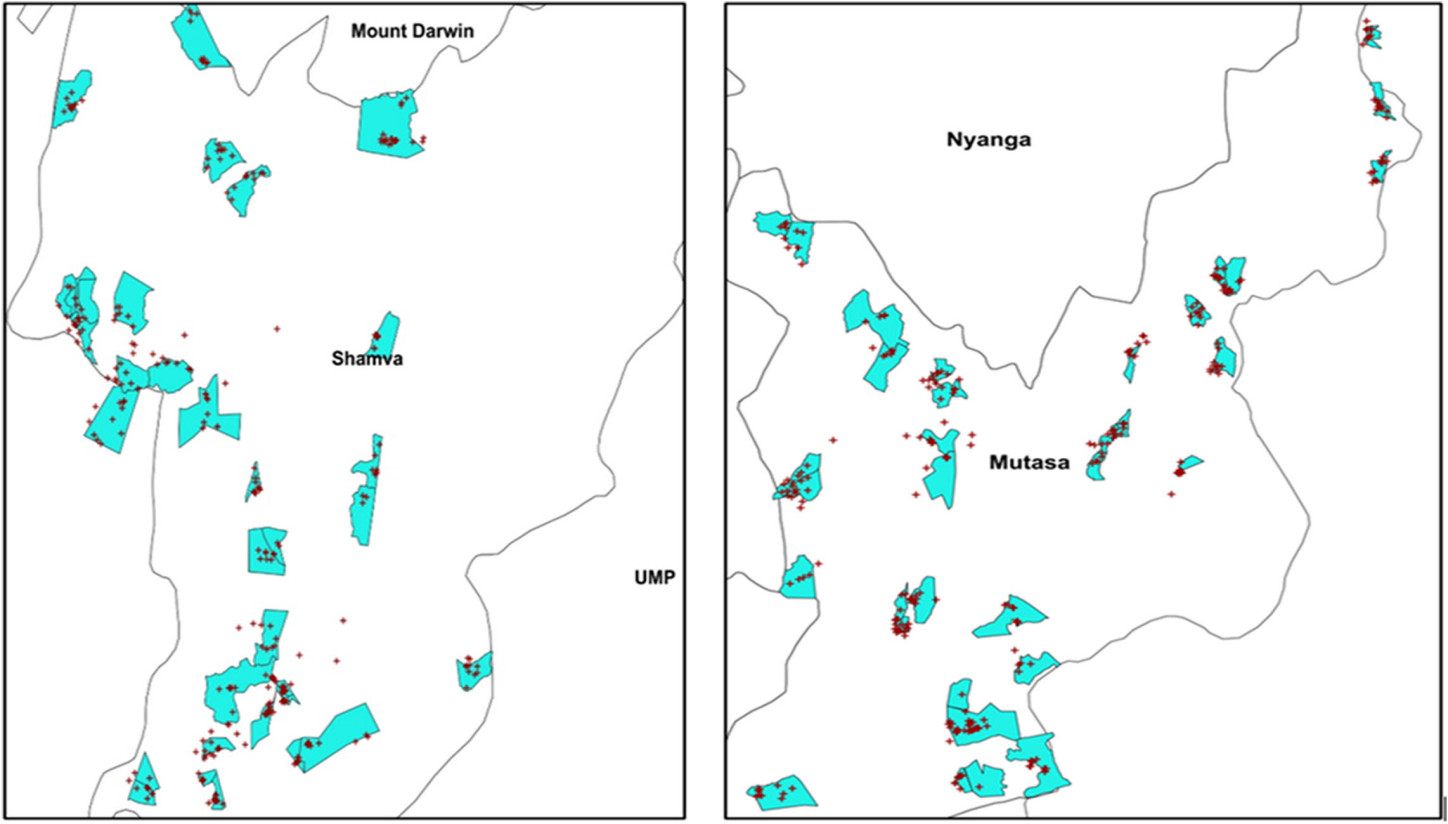
Maps of EAs (green) and selected households (red dots) in Shamva and Mutasa districts, Zimbabwe, based on GPS coordinates. (Lack of overlap is attributed to ellipsoidal height differences.)

At the EA level, all eligible households (defined as a unit of members eating food prepared from the same pot with at least one WRA and one child 6–59 months of age) were listed. Household lists provided the frame for the second stage, where systematic random sampling without replacement approach was used to select participating households. The location of each household within the EAs was determined using a global positioning system receiver (GPS) and verified through matched shape files (Figure 1). Only 10 households were selected in each of the sampled EAs using the formula:

$$\text{Phij}=\sum \frac{\text{AhMhi}}{\mathrm{Mhi}}\times \frac{\mathrm{nhi}}{\mathrm{Nhi}},$$

where:

- *Phij* is the selection probability of household number *j* in EA number *hi*
- *nhi* is the size of the household sample from EA number *hi*
- *Nhi* is the listed number of households in EA number *hi*

### 2.4 Tertiary sampling units (TSU)

In households where more than one set of eligible individuals were identified, one mother–child pair was randomly selected using the Kish grid method (Jabkowski, 2017). The degrees of freedom for variance components of a variable for which one measurement was made per household were as follows: 2 between‐district, 87 between‐EA within‐district, and 510 between‐household within‐district. Based on this approach, the study selected 1200 participants (600 WRA and 600 children aged 6–59 months). Recruitment was done at the household level, with participants then directed to the nearest health facility for data collection.

### 2.5 Inclusion and exclusion criteria

Women aged 15–49 years and children aged 6–59 months were eligible for the study. The exclusion criteria consisted of the following: chronically ill; outside the target age groups; diagnosed with chronic disease or long‐term illness requiring treatment; with acute or chronic infection; on regular prescribed medicine; regularly using dietary supplements including iodine, zinc, or selenium supplements; children with a physical disability that would affect either height or weight measurement, and if the mother–child pair were not resident in the study. The eligibility screening criteria were adapted from a similar survey conducted in Malawi (Phiri et al., 2019). Illnesses were either self‐reported and/or assessed by health practitioners.

### 2.6 Socioeconomic characteristics

Data were collected using face‐to‐face interviews at selected health facilities, observing national COVID‐19 guidelines. Enumeration was done with the help of trained enumerators who were resident nutritionists and/or other health workers in each district. A questionnaire adapted from ZDHS was used to collect household demographic data (ZIMSTAT, 2015). This questionnaire assessed socioeconomic (e.g., education level, marital status, income status, sources and expenditure), agricultural, water sanitation and hygiene (WASH), and infant and young child feeding (IYCF) practices.

### 2.7 Anthropometry

Weight, recumbent length, and height were measured according to WHO standard protocols (WHO, 2008). The anthropometry assessors were trained according to the WHO Training Course on Child Growth Assessment for infants (WHO, 2008). The weight of the children and WRA was measured to the nearest 0.01 kg using the 2 in 1 mother–child‐calibrated digital scale (Seca, Model 703). Length and height were measured to the nearest 0.1 cm using a standard wooden height board. Weight and length/height were measured twice, with a third measurement taken where there was inconsistency. The anthropometric indices, namely height‐for‐age *Z*‐score (HAZ), weight‐for‐height *Z*‐score (WHZ), and weight‐for‐age *Z*‐score (WAZ), were generated using the Emergency Nutrition Assessment software for SMART 2011 (Erhardt et al., 2015). WHO plausibility check was applied for standardization of anthropometric measurements as required for nutrition assessments of children aged 6–59 months (Ralston & Myatt, 2018; WHO, 2006). Birth weight was obtained from the infant's health cards. The standard formula, [weight (kg)]/[(height (m)]^2^, was used to calculate and classify body mass index (BMI) for WRA (WHO, 2011a). Body mass index below 18.5 was considered as underweight; 18.5–24.9, normal weight; 25.0–29.9, overweight; 30.0–34.9, obesity class I; 35.0–39.9, obesity class II; and above 40 defined as morbid obesity (WHO, 2011a). Maternal short stature was defined as a height below 145 cm (Sumarmi, 2016). In children aged 6–59 months old, wasting was defined as WHZ below −2 standard deviations (*SD*), stunting as HAZ below −2*SD*, underweight as WAZ below −2*SD*, and overweight as WHZ above +2*SD* (WHO, 2006). Low birth weight (LBW) was defined as a birth weight below 2.5 kg regardless of gestational age (WHO, 2011b).

### 2.8 Hemoglobin levels and anemia

Hemoglobin (Hb) testing is the primary method of anemia diagnosis (WHO, 2007). Hemoglobin (g/dl) concentration was measured in the field on a venous blood sample using a portable Hb801 HemoCue machine (HemoCue AB). The last drop of blood from venepuncture was drawn into a microcuvette and measured. Anemia was defined as Hb < 11 g/dl for children and Hb < 12 g/dl for WRA. Mild anemia corresponds to a Hb concentration of 10.0–10.9 g/dl for children under age 5 and 10.0–11.9 g/dl for nonpregnant WRA; moderate anemia corresponds to a level of 7.0–9.9 g/dl and severe anemia corresponds to a level less than 7.0 g/dl cases of anemia (WHO & Chan, 2011).

### 2.9 Data collection and management

Two teams of enumerators conducted data collection for approximately 12 days per district. Each team consisted of eight members and a team leader. All participants were assigned a unique numeric ID that was used in data capture forms and subsequent analyses to maintain anonymity. Questionnaire, anthropometry, and specimen data were collected via passcode‐protected tablets using KoboToolbox (Android v2022.1.2). Data were transferred daily to the central data processing server. The team leaders and data manager reconciled samples and data forms daily to check for participant totals and completeness of questionnaires.

### 2.10 Statistical analysis

Statistical analysis of data was performed using SPSS for Windows (v20). The normality of data was checked by Shapiro–Wilk test and Q–Q plots. The dependent variable was binary indicating the presence of anemia among WRA and children separately (not anemic = 0 and ANEMIC = 1). Association between the dependent variable and sociodemographic factors (age, sex, education level, marital status, and agricultural and WASH factors) and anthropometry indicators was evaluated using the Pearson Chi‐square test. Significant associations from the Pearson Chi‐square test at *p* < .05 were formulated into two multiple logistic regression analysis models: Model 1 on predictors of anemia in children 6–59 months old and Model 2 which evaluated the predictors of anemia in WRA.

## 3 RESULTS

Hemoglobin measurements were matched for 452 mother–child pairs from Shamva and Mutasa districts from which data are presented.

### 3.1 Socioeconomic characteristics

The median (±IQR) age for children was 29 ± 14 months with a proportional boy‐to‐girl ratio (Table 1) and that for women was 29 ± 12 years. The majority of WRA who participated in the study were married (91.8%) and had acquired secondary‐level education qualifications (67.3%). The median (±IQR) household size was 5.0 ± 2 with at least one child aged 6–59 months. The median (±IQR) household land size ownership was 2 ± 3 hectares with a greater proportion (55.8%) having ownership (as allocated by local rural authorities). Maize (*Zea mays*), 43%; groundnuts (*Arachis hypogaea L*.), 29%; cow peas (*Vigna unguiculata*), 27%; and sweet potatoes (*Ipomoea batatas*), 25% were the commonly grown crops for consumption. All households had livestock, with combinations constituting 13.3% cows (*Bos taurus*), 0.7% sheep (*Ovis aries*), 34.6% goats (*Capra hircus*), and 62.7% poultry (*Gallus gallus domesticus*). Only a few households (10.0%) earned a monthly household income of United States Dollars (USD) >220 adequate to meet the total consumption poverty line (TCPL) set at USD 63.50 per person as of August 2021 (ZIMSTAT, 2021) (Table 1). The main sources of household income were self‐employment (32.7%), formal employment (20.9%), casual labor (18.2%), and cash crop farming (10.5%).

**TABLE 1:** Sociodemographic characteristics of children aged 6–59 months in rural Zimbabwe by anemia status.

Variable	Total, *n* (% of category)	Nonanemic, *n* (%)^a^	Anemic, *n* (%)^b^	*p*‐value^†^
Sex				
Boy	229 (50.7)	151 (65.9)	78 (34.1)	.040*
Girl	223 (49.3)	167 (74.9)	56 (25.1)	
Age group (months)				
6–8	24 (5.3)	17 (70.8)	7 (29.2)	.810
9–11	27 (6.0)	20 (74.1)	7 (25.9)	
12–17	71 (15.7)	52 (73.2)	19 (26.8)	
18–23	76 (16.8)	57 (75.0)	19 (25.0)	
24–35	94 (20.8)	67 (71.3)	27 (28.7)	
36–47	91 (20.1)	60 (65.9)	31 (34.1)	
48–59	69 (15.3)	45 (65.2)	24 (34.8)	
Number of children under 5 years in household				
1	335 (74.1)	232 (69.3)	103 (30.7)	.412
>1	117 (25.9)	86 (73.5)	31 (26.5)	
Household size				
≤4	186 (41.2)	129 (69.4)	57 (30.6)	.754
>4	266 (58.8)	189 (71.1)	77 (28.9)	
Monthly income (USD)				
<10	12 (2.7)	10 (83.3)	2 (16.7)	.233
10–50	172 (38.1)	112 (65.1)	60 (34.9)	
51–110	144 (31.9)	101 (70.1)	43 (29.9)	
120–210	79 (17.5)	61 (77.2)	18 (22.8)	
>220	45 (10.0)	34 (75.6)	11 (24.4)	
Agricultural land ownership				
No	200 (44.2)	140 (70.0)	60 (30.0)	.918
Yes	252 (55.8)	178 (70.6)	74 (29.4)	
Livestock ownership				
Cows				
2	450 (99.6)	317 (70.4)	133 (29.6)	>.999
>2	2 (0.4)	1 (50.0)	1(50.0)	
Chicken /poultry				
1	176 (38.9)	123 (69.9)	53 (30.1)	.916
>1	276 (61.1)	195 (70.7)	81 (29.3)	
Unimproved dug wells as a source of drinking water				
No	366 (81.0)	268 (73.2)	98 (26.8)	.006*
Yes	86 (19.0)	50 (58.1)	36 (41.9)	
Location of water source				
Off‐premise (elsewhere)	340 (75.2)	237 (69.7)	103 (30.3)	.643
In‐house (own dwelling)	20 (4.4)	16 (80.0)	4 (20.0)	
On‐premise (own yard/plot)	92 (20.4)	65 (70.7)	27 (29.3)	
Insufficient water in past month				
No	341 (75.4)	245 (72.1)	96 (27.9)	.119
Yes	111 (24.6)	73 (65.8)	38 (34.2)	
Treatment of drinking water				
No	407 (90.0)	283 (69.5)	124 (30.5)	.304
Yes	45 (10.0)	35 (77.8)	10 (22.2)	
Toilet facility				
No	7 (1.5)	5 (71.4)	2 (28.6)	>.999
Yes	445 (98.5)	313 (70.3)	132 (29.7)	
Toilet facility shared with other households				
No	315 (69.7)	221 (70.2)	94 (29.8)	.911
Yes	137 (30.3)	97 (70.8)	40 (29.2)	
Toilet facility on‐premise				
No	273 (60.4)	206 (75.5)	67 (24.5)	.004*
Yes	179 (39.6)	112 (62.6)	67 (37.4)	
Overall anemia prevalence	100 (452)	318 (70.4)	134 (29.6)	

Abbreviations: HAZ, height‐for‐age *Z*‐score; *SD*, Standard deviation; WAZ, weight‐for‐age *Z*‐score; WHZ, weight‐for‐height *Z*‐score.

^a^ Hb level ≥ 11 g/dl.

^b^ Hb level < 11 g/dl.

*Significant at *p* < .05; †*p* value from Pearson's χ^2^ test.

The location of the water source was off‐premise for the majority (75.2%) of households (Table 1). Most dug wells (81%) were not improved, that is, not lined, covered, and fitted with a secure water lifting device, such as a pump or windlass (WHO, 2020b). A minority (10.0%) of households treated their drinking water by boiling or chlorination before consumption. Insufficient water supply 30 days prior to the survey was reported by a few (24.6%) of the households. The greater proportion (98.5%) of the households had access to toilet facilities constituting pit latrine (82.9%), flush (8.1%), and composting (7.5%), with a minority (30.3%) sharing toilet facilities (Table 1).

Health‐seeking behavior upon child illness was high at 92.9%, with the remainder not seeking treatment. Treatment advice was mostly sought from the public health sector (88.5%) comprising of Government (hospitals, health center/post, and community health workers), followed by the private sector (3.5%), and other sources (shops, traditional practitioners, and market, wandering drug seller) were the least (0.6%). Government health centers constituted 76.8% of the visited public health sections and the majority of these were within a distance of 1.5 km (66.2%) from households, and the furthest distance (>10 km) was reported by 5.1% of the households.

### 3.2 Nutritional and health status

#### 3.2.1 Children 6–59 months old

Almost a third (27.8%) of the children were stunted, 2.6% were wasted, and 13.7% were underweight (Table 2). The proportion of stunting (28.9% vs. 26.6%; *p* = .665), wasting (2.8% vs. 2.4%; *p*= > .999), and underweight (17.1% vs. 10.4%; *p* = .053) was higher in boys as compared to girls, respectively. Children below 24 months of age had lower proportions of stunting (25.1% vs. 29.9%; *p* = .279), wasting (2.1% vs. 3.0%; *p* = .761), and underweight (11.2% vs. 15.7%; *p* = .211) compared to children 24 months and older, respectively. The prevalence of breastfeeding was high with 99.8% of children having been ever breastfed; the rate of exclusive breastfeeding was high (68.3%) and that of LBW was low (9.3%). Disease prevalence 2 weeks preceding the survey date indicated diarrhea had the lowest prevalence (26.8%), followed by fever (33.6%), and respiratory infection had the highest prevalence (38.9%). Vitamin A supplementation was high (76.8%), while deworming (29.4%) and micronutrient powder (MNP) supplementation (14.6%) coverage were low (Table 2). Micronutrient powder supplementation was highest in the 9–11 months age group and lowest in the 36–47 months age group with up to 6% of the former and 20.8% of the latter not supplemented.

**TABLE 2:** Nutritional status and morbidities in children aged 6–59 months in rural Zimbabwe by anemia status.

Variable	Total, *n* (% of category)	Nonanemic, *n* (%)^a^	Anemic, *n* (%)^b^	*p*‐value^†^
Stunted (HAZ)				
Below −2*SD*	118 (27.8)	84 (71.2)	34 (28.8)	.726
−2SD and above	307 (72.2)	213 (69.4)	94 (30.6)	
Wasting (WHZ)				
Below −2*SD*	11(2.6)	8 (72.7)	3 (27.3)	>.999
−2*SD* and above	414 (97.4)	290 (70.0)	124 (30.0)	
Underweight (WAZ)				
Below −2*SD*	61 (13.7)	42 (68.9)	19 (31.1)	.763
−2*SD* and above	383 (86.3)	272 (71.0)	111 (29.0)	
Low Birth Weight (kg)				
<2.5	42 (9.3)	32 (76.2)	10 (23.8)	.321
≥2.5	402 (88.9)	282 (70.1)	120 (29.9)	
Exclusive breastfeeding				
No	143 (31.7)	103 (72.0)	40 (28.0)	.658
Yes	308 (68.3)	214 (69.5)	94 (30.5)	
Vitamin A supplementation				
No	105 (23.2)	79 (73.7)	26 (26.3)	.182
Yes	347 (76.8)	239 (68.9)	108 (31.1)	
MNP supplementation				
No	386 (85.4)	267 (69.1)	119 (30.9)	.432
Yes	66 (14.6)	51 (77.3)	15 (22.7)	
Deworming				
No	319 (70.6)	229 (74.0)	90 (26.0)	.510
Yes	133 (29.4)	89 (66.9)	44 (33.1)	
Diarrhea				
No	331 (73.1)	236 (71.3)	95 (28.7)	.486
Yes	121 (26.8)	82 (67.8)	39 (32.2)	
Fever				
No	300 (66.4)	209 (69.7)	91 (30.3)	.665
Yes	152 (33.6)	109 (71.7)	43 (28.3)	
Respiratory tract infection				
No	276 (61.1)	197 (71.4)	79 (28.6)	.598
Yes	176 (38.9)	121 (68.8)	55 (31.2)	

Abbreviations: HAZ, height‐for‐age *Z*‐score; *SD*, Standard deviation; WAZ, weight‐for‐age *Z*‐score; WHZ, weight‐for‐height *Z*‐score.

^a^ Hb level ≥ 11 g/dl.

^b^ Hb level < 11 g/dl.

†*p* value from Pearson's χ^2^ test.

#### 3.2.2 Women of reproductive age

Most of the women (99.3%) had adequate stature with only 0.7% being of short stature (Table 3). The median (±IQR) height was 159 ± 8 cm. Based on BMI, a few women (6.9%) were underweight, the majority (55.8%) had normal weight, and the proportions of overweight, class I, class II, and morbid obesity were 26.3%, 7.8%, 2.0%, and 1.1%, respectively.

**TABLE 3:** Maternal demographic factors and nutritional status of children 6–59 months old in rural Zimbabwe by anemia status.

Variable	Total, *n* (% of category)	Nonanemic, *n* (%)^a^	Anemic, *n* (%)^b^	*p* value^†^
Age (years)				
15–19	25 (5.5)	17 (68.0)	8 (32.0)	.747
20–24	102 (22.6)	75 (73.5)	27 (26.5)	
25–29	105 (23.2)	73 (69.5)	32 (30.5)	
30–34	79 (17.5)	57 (72.2)	22 (27.8)	
35–39	76 (16.8)	55 (72.4)	21 (27.6)	
40–44	47 (10.4)	28 (59.6)	19 (40.4)	
45–49	18 (4.0)	13 (72.2)	5 (27.8)	
Reproductive age				
≤35	316 (69.9)	224 (70.9)	92 (29.1)	.737
>35	136 (30.1)	94 (69.1)	42 (30.9)	
Mothers' marital status				
Married monogamy	385 (85.2)	270 (70.1)	115 (29.9)	.885
Married polygamy	30 (6.6)	21 (70.0)	9 (30.0)	
Separated/divorced	29 (6.4)	22 (75.9)	7 (24.1)	
Single/never married	4 (0.9)	3 (75.0)	1 (25.0)	
Widowed	4 (0.9)	2 (50.0)	2 (50.0)	
Mothers' education status				
Tertiary	3 (0.7)	3 (100)	0 (0)	.181
Advanced level	6 (1.3)	3 (50.0)	3 (50.0)	
Ordinary level	304 (67.3)	220 (72.4)	84 (27.6)	
Primary	131 (29.0)	85 (64.9)	46 (35.1)	
None	8 (1.8)	7 (87.5)	1 (12.5)	
Body Mass Index (kg/m^2^)				
<18.5	31 (6.9)	21 (67.7)	10 (32.3)	.839
≥18.5	421 (93.1)	297 (70.5)	124 (29.5)	
Height (cm)				
<145	3 (0.7)	2(66.7)	1 (33.3)	>.999
≥145	449 (99.3)	316 (70.4)	133 (29.6)	
Anemia status				
Not anemic	371 (82.1)	273 (85.8)	98 (26.4)	.002*
Anemic	81 (17.9)	45 (55.6)	36 (44.4)	
Overall anemia prevalence	*n* = 452	318 (70.4)	134 (29.6)	

HAZ, height‐for‐age *Z*‐score; *SD*, Standard deviation; WAZ, weight‐for‐age *Z*‐score; WHZ, weight‐for‐height *Z*‐score.

^a^ Hb level ≥ 11 g/dl.

^b^ Hb level < 11 g/dl.

*Significant at *p* < .05; †*p* value from Pearson's χ^2^ test.

### 3.3 Hemoglobin levels and anemia

**FIGURE 2:**
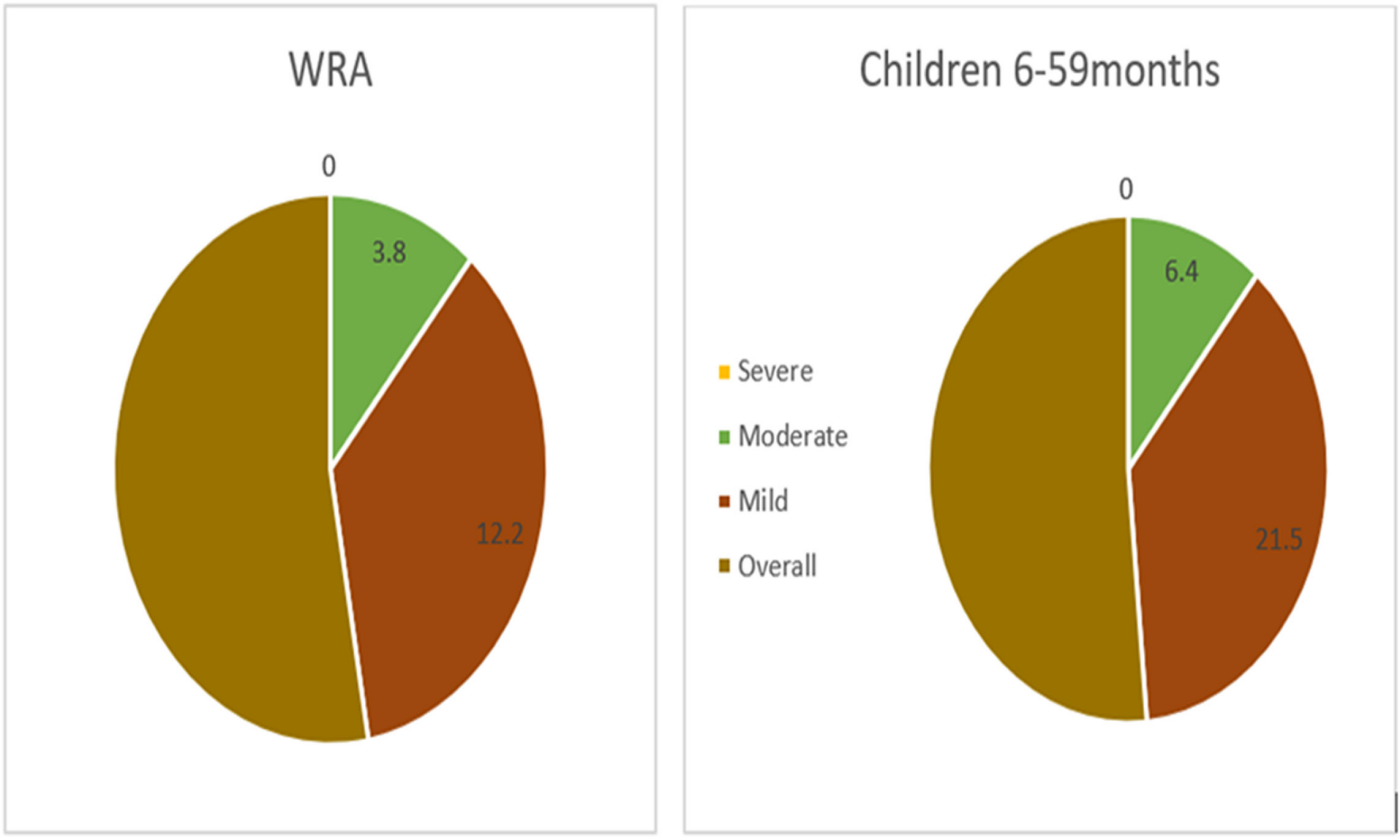
Types of anemia in children aged 6–59 months and WRA (overall Anemia: Hb < 11 g/dl for children and Hb < 12 g/dl for WRA; mild anemia; Hb of 10.0–10.9 g/dl for children and 10.0–11.9 g/dl WRA; moderate anemia; Hb of 7.0–9.9 g/dl; and severe anemia Hb <7.0 g/dl).

The overall prevalence of anemia in WRA was 17.9% with a median (±IQR) Hb level of 13.4 ± 1.8 g/dl and 29.6% in children with a median (±IQR) Hb level of 11.7 ± 1.5 g/dl. There were no severe cases of anemia in both WRA and children, only moderate and mild cases were observed (Figure 2).

### 3.4 Predictors of anemia in children and WRA

Pearson Chi‐square test indicated that the male sex (*p* = .040), unimproved dug wells as a source of drinking water (*p* = .006), on‐premise toilet facility (*p* = .004) (Table 1), and maternal anemia (*p* = .002) (Table 3) were positively associated with anemia in children, whereas in WRA, unimproved dug wells (*p* < .001) as a source of drinking water, on‐premise toilet facility (*p* = .060), and lack of agricultural land ownership (*p* = .007) (Table 4) were positively associated with anemia.

**TABLE 4:** Sociodemographic and nutritional status of WRA in rural Zimbabwe anemia status.

Variable	Total, *n* (% of category)	Nonanemic, *n* (%)^a^	Anemic, *n* (%)^b^	*p*‐value^†^
Height (cm)				
<145	3 (0.7)	3 (100)	0 (0)	.636
≥145	449 (99.3)	368 (82.0)	81 (18.0)	
Body Mass Index (kg/m^2^)				
<18.5	31 (6.9)	27 (87.1)	4 (12.9)	.488
≥18.5	421(93.1)	344 (81.7)	77 (18.3)	
Age (years)				
15–19	25 (5.5)	21 (84.0)	4 (16.0)	.175
20–24	102 (22.6)	84 (82.4)	18 (17.6)	
25–29	105 (23.2)	88 (83.8)	17 (16.2)	
30–34	79 (17.5)	66 (83.5)	13 (16.5)	
35–39	76 (16.8)	54 (71.1)	22 (28.9)	
40–44	47 (10.4)	42 (89.4)	5 (10.6)	
45–49	18 (4.0)	16 (88.9)	2 (11.11)	
Reproductive Age (years)				
≤35	316 (69.9)	262 (82.9)	54 (17.1)	.505
>35	136 (30.1)	109 (80.1)	27 (19.9)	
Mothers' marital status				
Married monogamy	385 (85.2)	312 (81.0)	73 (19.0)	.444
Married polygamy	30 (6.6)	25 (83.3)	5 (16.7)	
Separated/divorced	29 (6.4)	27 (93.1)	2 (6.9)	
Single /never married	4 (0.9)	4 (100)	0 (0.0)	
Widowed	4 (0.9)	3 (75.0)	1 (25.0)	
Mothers' education status				
Tertiary	3 (0.7)	2 (66.7)	1 (33.3)	.638
Advanced level	6 (1.3)	4 (66.7)	2 (33.3)	
Ordinary level	304 (67.3)	254 (83.6)	50 (16.4)	
Primary	131 (29.0)	105 (80.2)	26 (19.8)	
None	8 (1.8)	6 (75.0)	2 (25.0)	
Number of children under 5 years in household				
1	335 (74.1)	276 (82.4)	59 (17.6)	.780
>1	117 (25.9)	95 (81.2)	22 (18.8)	
Household size				
≤4	186 (41.2)	151 (81.2)	35 (18.8)	.709
>4	266 (58.8)	220 (82.7)	46 (17.3)	
Monthly Income (USD)				
<10	12 (2.7)	9 (75.0)	3 (25.0)	.381
10–50	172 (38.1)	136 (79.1)	36 (20.9)	
51–110	144 (31.9)	122 (84.7)	22 (15.3)	
120–210	79 (17.5)	69 (87.3)	10 (12.7)	
>220	45 (10.0)	35 (77.8)	10 (22.2)	
Agricultural land ownership				
No	200 (44.2)	153 (76.5)	47 (23.5)	.007*
Yes	252 (55.8)	218 (86.5)	34 (13.5)	
Livestock ownership				
Cows				
2	450 (99.6)	370 (82.2)	80 (17.8)	.327
>2	2 (0.4)	1 (50.0)	1 (50.0)	
Chicken /poultry				
1	176 (38.9)	138 (78.4)	38 (21.6)	.131
>1	276 (61.1)	233 (84.4)	43 (15.6)	
Unimproved dug wells as a source of drinking water				
No	366 (81.0)	313 (85.5)	53 (14.5)	<.001*
Yes	86 (19.0)	58 (67.4)	28 (32.6)	
Location of water source				
Off‐premise	340 (75.2)	285 (83.8)	55 (16.2)	.076
In‐house	20 (4.4)	13 (65.0)	7 (35.0)	
On‐premise	92 (20.4)	73 (79.3)	19 (20.7)	
Insufficient water in past month				
No	341 (75.4)	279 (81.8)	62 (18.2)	.907
Yes	111 (24.6)	92 (82.9)	19 (17.1)	
Treatment of drinking water				
No	407 (90.0)	335 (82.3)	72 (17.7)	.838
Yes	45 (10.0)	36 (80.0)	9 (20.0)	
Toilet facility				
No	7 (1.5)	5 (71.4)	2 (28.6)	.614
Yes	445 (98.5)	366 (82.2)	79 (17.8)	
Toilet facility shared with other households				
No	315 (69.7)	260 (82.5)	55 (17.5)	.790
Yes	137 (30.3)	111 (81.0)	26 (19.0)	
Toilet facility on‐premise				
No	273 (60.4)	232 (85.0)	41 (15.0)	.060*
Yes	179 (39.6)	139 (77.7)	40 (22.3)	
Prevalence of anemia (WRA)	*n* = 452	371 (82.1)	81 (17.9)	

^a^ Hb level ≥ 12 g/dl.

^b^ Hb level < 12 g/dl.

*Significant at *p* < .05; †*p* value from Pearson's χ^2^ test.

Multiple regression analysis showed significant associations of anemia in children with having an anemic mother (*p* = .007) and being a male child (*p* = .029), while in WRA, anemia was significantly associated with using unimproved dug wells as a source of drinking water (*p* = .001) and lack of agricultural land ownership (*p* = .009) (Table 5). Children whose mothers were anemic were 2.02 times more likely to be anemic compared to those whose mothers were nonanemic (OR = 2.02; 95% CI 1.21–3.37; *p* = .007) and the boy child was 0.63 times more likely to be anemic than the girl child (OR = 0.63; 95% CI 0.41–0.95; *p* = .029). Women from households with unimproved dug wells as sources of drinking water were 0.36 times more likely to be anemic compared to those drinking water from improved wells (OR = 0.36; 95% CI 0.20–0.66; *p* = .001), and women who did not own agricultural land were 0.51 times more likely to be anemic than those owning agricultural land (OR = 0.51; 95% CI 0.31–0.85; *p* = .009).

**TABLE 5:** Predictors of anemia among children aged 6–59 months and WRA from rural Zimbabwe.

Variable	*B*	*SE*	*p* value^†^	OR	95% CI	
					Lower	Upper
Children (6–59 months) – Model 1						
Maternal anemia (yes = 0 no = 1)	0.70	0.26	.007⁎	2.02	1.21	3.37
Being a boy (female =0 male = 1)	−0.47	0.21	.029⁎	0.63	0.41	0.95
Unimproved dug well as a water source (improved dug well = 0; unimproved = 1)	−0.38	0.28	.181	0.69	0.39	1.19
On‐premise toilet facility (off‐premise = 0; on‐premise = 1)	−0.44	0.23	.060	0.65	0.41	1.02
Women (15–49 years) – Model 2						
Unimproved dug well as a water source (improved = 0; unimproved = 1)	−1.02	0.31	.001⁎	0.36	0.20	0.66
On‐premise toilet facility (off‐premise = 0; on‐premise = 1)	−0.03	0.28	.914	0.97	0.56	1.69
Lack of agricultural land ownership (land ownership = 0; lack of ownership = 1)	−0.67	0.25	.009⁎	0.51	0.31	0.85

*Significant at *p* < .05. †*p* value from multiple logistic regression analysis; *p* < .10 was used as a cut‐off point to retain variables in the regression model.

## 4 DISCUSSION

The study sought to determine the prevalence of anemia in WRA and children aged 6–59 months as well as potential predictors. According to the WHO classification, our results show that anemia is of moderate public health significance in children (20%–39%) and mild among WRA (5%–19%) (WHO, 2012) in the selected districts. Thus, we speculate that anemia is potentially a public health issue of national relevance. The prevalence of anemia in the two districts was lower compared to the 2015 ZDHS national survey prevalence of 37% in children and 27% in WRA, respectively (ZIMSTAT, 2015). This might be alluded to the commencement in October 2017 of a MNP supplementation program to children aged 6–23 months in rural districts by the Ministry of Health and Child Care and UNICEF Zimbabwe. Micronutrient powder supplementation is known to significantly reduce the prevalence of anemia and iron deficiency anemia, and improve Hb levels (Mahfuz et al., 2016; Salam et al., 2013). In addition, this can also be due to a higher exclusive breastfeeding rate (68.3%) noted in the current study compared to 2015 when it was reported to be 48% (ZIMSTAT, 2015) since breastmilk is an important source of micronutrients (Allen et al., 2006). Higher rates of mild anemia were indicated compared to moderate anemia, and there were no cases of severe anemia in both women and children. This indicates reduced severity of anemia within the selected district populations. Median Hb concentrations were comparable to other studies conducted in the region; Togo and sub‐Saharan African countries (Nambiema et al., 2019; Worku et al., 2022).

### 4.1 Predictors of anemia in children 6–59 months old (model 1)

Our results show that gender was significantly associated with anemia risk in children. Boys were more likely to be anemic than girls, which was consistent with previous studies in Ethiopia (Melku et al., 2018), Togo (Nambiema et al., 2019), and in Boricha Woreda, Southern Ethiopia (Yoseph & Beyene, 2020). The ZDHS 2015 indicated a small variation by gender (38% of boys were anemic compared with 36% of girls) while other studies in rural Tanzania have found the opposite (Mrimi et al., 2022). The higher prevalence in boys could be related to the higher growth rate in boys resulting in a greater need for iron by the body, which might not be met by the diet (Silla et al., 2013). This could be explained by the growth, development, and maturation stages after birth, including the postnatal spurt followed by the transition from infancy to childhood (Hermanussen, 2016). The growth spurt coupled with termination of lactation by about age 2 years poses a high nutrient demand which may be difficult to meet particularly among boys when compared to girls at age 6–59 months (Khara et al., 2018). Therefore, there is a need to emphasize differences in diet/nutrient requirements based on sex within the generic “one size fits all” recommendations currently being employed in infant and young child feeding (IYCF) education and counseling sessions in health centers.

In the current study, the occurrence of anemia in the mothers was a significant positive predictor of anemia in children. Compared to nonanemic mothers, anemic mothers were significantly more likely to have anemic children. These findings were consistent with results from a multilevel analysis conducted for Southern Africa covering Malawi, Mozambique, and Namibia (Ntenda et al., 2018). This can be attributed to low levels of essential minerals such as iron, zinc, and folate, as well as vitamins A and B12, in the breast milk of the anemic mother, which could also affect the Hb level of the breastfeeding child (Wang et al., 2015). Furthermore, it can potentially be a result of the depletion of iron stores due to successive pregnancies and lactation resulting in iron deficiency anemia (Cane et al., 2022; Cardoso et al., 2012). Additionally, it might be due to that mothers and their children share similar socioecological environments. Thus, their dietary patterns, disease profiles or exposures, and quality of life may be similar (Ntenda et al., 2018), suggestive of intergenerational transmission of poor maternal socioeconomic, health, and nutritional status (Martorell & Zongrone, 2012). Therefore, interventions that focus on improving preconceptual and maternal nutritional status may be warranted. For example, the government‐supported iron and folate supplementation targeted for pregnant women in Zimbabwe may need to be extended to cover all WRA (15–49 years). In addition, considering the evidence on the benefits of multiple micronutrient supplementation (MMS) for pregnant women in some settings (Christian, 2015), the approach should be considered in low socioeconomic settings like Zimbabwe to ensure positive impacts on anemia prevalence.

### 4.2 Predictors of anemia in WRA (model 2)

Our results showed that anemia risk in women was positively associated with lack of agricultural land ownership, with those owning land being at a lower risk of being anemic compared to those without land ownership. This can be explained by improved dietary diversity and food security through diversified crop production (Kristjanson et al., 2010). Lack of access to land may predispose populations to food insecurity which consequently increases susceptibility to anemia (FAO, 2000; WFP/FAO, 2014). This is, however, contrary to a similar study in India, which found that women who had their own agricultural land suffered more from anemia compared to those who did not own any agricultural land. This was explained by time trade‐offs between women's participation in agriculture and food preparation in the household which negatively impacted their nutritional status (Jana et al., 2022). The recommendation is for government to devote itself to food security programs that promote land ownership. However, literature is limited on the linkages between agricultural land ownership and anemia. Further research could help clarify the linkage so as to inform livelihood programs.

Results from the current study indicated that unimproved dug wells as a source of drinking water were a positive predictor of anemia in WRA, with those relying on unimproved dug wells being at a greater risk of anemia than those using improved wells. This result was consistent with a study undertaken in Nepal (Gautam et al., 2019), India, and Bangladesh (Jana et al., 2022) where women in households using dug wells as sources of drinking water were significantly associated with an increased risk of developing anemia. In the case of Bangladesh, it was partly attributable to fluoride and arsenic in groundwater (Del Bello, 2020; Jana et al., 2022). In some contexts, it may be due to intestinal worm infections (Chelkeba et al., 2022; WHO, 2022) which cause gut blood losses, malabsorption of nutrients, loss of appetite, and anemia due to loss of iron and impaired protein metabolism (Pullan et al., 2014). However, in other contexts it might be due to higher incidences of diarrheal diseases and upper respiratory tract infections (Marakwet & SNV, 2018). Consistent findings were observed in Peru (Mougenot et al., 2020) and Nepal (Coffey & Geruso, 2015) where safe drinking water from improved dug wells was associated with reduced anemia prevalence. While it is expected that access to improved water and sanitation facilities lowers anemia prevalence, in the current study, on‐premise toilet facilities increased the prevalence of anemia. Similar mixed‐direction findings were observed in previous studies in Zimbabwe (SHINE, 2015) and other surveys (Yu et al., 2019) which showed that access to improved water and sanitation does not consistently predict lower anemia prevalence. Despite the heterogeneity, hygiene interventions including hand washing with soap and improved water and sanitation can reduce the disease risk associated with anemia (Borghi et al., 2002; Ejemot‐Nwadiaro et al., 2015; Ejemot‐Nwadiaro et al., 2008; WHO, 2022). Therefore, promoting policies and practices that strengthen access to improved WASH should still be considered for reducing anemia prevalence. Additionally, the promotion of regular water consumption as a source of minerals is warranted since iron in groundwater provides a good source of absorbable iron for humans and can contribute to optimal iron and Hb status among populations (Choudhury et al., 2022).

### 4.3 Limitations of study

The cross‐sectional nature of the present study limits the ability to make inferences on causation. The current study is exploratory and a substudy from a larger trial with preplanned outcomes. Tests of statistical significance should be interpreted bearing this in mind, as well as the possibility of false positives (Type 1 error) due to multiple comparisons. Adjustment for multiple comparisons was not conducted since this was an exploratory study and we wanted to avoid false negatives (Type 2 error). Additional dedicated studies are needed to confirm the results. The study did not differentiate the types of anemia, and sample size would be a limiting factor for greater refinement of several related research questions. Nevertheless, the present study contributes to the body of knowledge that shows the associations between maternal and child anemia, and environmental and nutritional characteristics in vulnerable populations.

## 5 CONCLUSIONS

The current study showed that the prevalence of anemia was moderate, mainly affecting male children; with maternal anemia being a positive predictor of child anemia. Unimproved dug wells as a source of drinking water and lack of ownership of agricultural land were positive predictors of anemia in WRA. Interventions that focus on improving preconceptual and maternal nutritional status, MNP supplementation, access to improved water and sanitation, and agricultural land ownership may be important strategies to reduce anemia in vulnerable populations from low‐ and lower middle‐income countries.

## ETHICAL APPROVAL

The funding bodies had no influence on the study design, data collection, analysis or interpretation of the data, writing of the manuscript, or the decision to submit the manuscript for publication. The study was conducted in line with the Declaration of Helsinki, and ethical approval was obtained from the Institutional Review Boards (IRBs) of the University of Nottingham (Reference #446–1912) and the Medical Research Council of Zimbabwe (MRCZ/A/2575 & MRCZ/A/2664). Permission to collect data in communities was obtained through consultative engagement with local government officials and the ministry of health at the provincial, district, clinic, and village levels. Written informed consent was obtained from all participants prior to the commencement of data collection.

## Data Availability

The data that support the findings of this study are available on request from the corresponding author. The data are not publicly available due to privacy/ethical restrictions.
